# Breast Cancer and Metabolic Dysfunction-Associated Steatotic Liver Disease

**DOI:** 10.3390/ijms27114919

**Published:** 2026-05-29

**Authors:** Damaris G. Nieva-Ramírez, David Luna-Pérez, Misael Uribe, Natalia Nuño-Lámbarri

**Affiliations:** 1 Traslational Research Unit, Medica Sur Clinic & Foundation, Puente de Piedra 150, Toriello Guerra Tlalpan, Mexico City Z.C. 14050, Mexico; damaris.nievar@alumno.buap.mx (D.G.N.-R.); david.lunaper@alumno.buap.mx (D.L.-P.); 2 School of Medicine, Benemérita Universidad Autónoma de Puebla, 13 Sur 2702, Los Volcanes, Puebla Z.C. 72420, Mexico; 3 Obesity and Digestive Diseases Unit, Medica Sur Clinic & Foundation, Puente de Piedra 150, Toriello Guerra Tlalpan, Mexico City Z.C. 14050, Mexico; muribe@medicasur.org.mx; 4 Department of Surgery, Faculty of Medicine, The National Autonomous University of Mexico (UNAM), Mexico City Z.C. 04360, Mexico

**Keywords:** hepatokines, lipid metabolism, inflammation, breast neoplasms, liver

## Abstract

Breast cancer remains the most frequently diagnosed malignancy among women worldwide, while metabolic dysfunction-associated steatotic liver disease (MASLD) represents the leading cause of chronic liver disease, reflecting a global burden of metabolic dysfunction. Increasing evidence suggests that MASLD is associated with breast cancer development and progression; however, whether this relationship reflects an independent effect of hepatic metabolic dysfunction or the broader metabolic environment remains uncertain. This review synthesizes current epidemiological, clinical, and mechanistic data linking hepatic metabolic dysfunction to breast carcinogenesis. Population-based studies consistently demonstrate an association between hepatic steatosis and increased breast cancer incidence, particularly in postmenopausal and metabolically vulnerable populations, as well as poorer oncological outcomes. Mechanistically, MASLD promotes a systemic pro-tumorigenic environment through interconnected pathways, including insulin resistance, hormonal dysregulation with increased estrogen bioavailability, chronic inflammation, oxidative stress, lipid metabolic reprogramming, and gut–liver axis disruption. Hepatokines, particularly fibroblast growth factor 21 (FGF21), emerge as key mediators of tumor progression and potential biomarkers of metabolic vulnerability, while Fetuin-A and angiopoietin-like protein 8 (ANGPTL8) further support the liver’s endocrine role in oncogenic signaling. Preclinical evidence highlights fatty acid oxidation as a metabolic dependency in aggressive breast cancer subtypes, suggesting novel therapeutic targets. Despite consistent associations, causality remains unproven. Future prospective studies are needed to determine whether targeting metabolic dysfunction can improve breast cancer prevention and outcomes.

## 1. Introduction

Breast cancer remains the most frequently diagnosed malignancy among women worldwide, with an annual incidence exceeding two million new cases and accounting for approximately 11–12% of all global cancer diagnoses [1]. Although its prevalence is higher in high-income countries, more than 60% of cases and nearly 70% of related deaths occur in low- and middle-income regions, where mortality rates are disproportionately elevated due to delayed diagnosis and limited access to optimal treatment strategies. The incidence of breast cancer increases with age, with a median age at diagnosis of 63 years, and it is more commonly observed in postmenopausal women [2,3].

Established risk factors include non-modifiable elements such as age, family history, and genetic mutations (e.g., *BRCA1/2*), as well as modifiable factors closely linked to metabolic health, including obesity, physical inactivity, and exposure to hormonal therapies [4]. Prognosis is largely determined by the stage at diagnosis and molecular subtype. While approximately 70–80% of early-stage cases are potentially curable, metastatic disease remains incurable, with median survival ranging from approximately one year in metastatic triple-negative breast cancer to up to five years in luminal and HER2-positive subtypes [5].

In parallel, metabolic dysfunction-associated steatotic liver disease (MASLD), formerly known as nonalcoholic fatty liver disease (NAFLD), has emerged as the most prevalent form of chronic liver disease globally [6]. MASLD is defined by the presence of hepatic steatosis in the absence of significant secondary causes and is intrinsically associated with cardiometabolic abnormalities such as obesity, type 2 diabetes mellitus, dyslipidemia, and hypertension [7,8]. Its global prevalence is estimated at 38.77% in the general population, rising to over 50.7% among individuals with overweight or obesity, underscoring its substantial public health burden [9,10].

MASLD is increasingly recognized as a systemic disorder reflecting global metabolic dysfunction, characterized by insulin resistance, chronic low-grade inflammation, and hormonal dysregulation. Beyond hepatic involvement, accumulating evidence has linked MASLD to an increased risk of several extrahepatic malignancies, including breast cancer, suggesting that hepatic metabolic dysfunction may contribute to a broader systemic metabolic and inflammatory milieu associated with carcinogenic processes.

## 2. Methods

A comprehensive literature search was conducted to identify relevant studies exploring the relationship between metabolic dysfunction-associated steatotic liver disease (MASLD) and breast cancer. The search was performed across three major electronic databases: PubMed/MEDLINE, Scopus, and Web of Science. The search strategy combined Medical Subject Headings (MeSH) and free-text terms related to MASLD and breast cancer, including but not limited to: “MASLD”, “NAFLD”, “metabolic dysfunction-associated fatty liver disease”, “breast cancer”, “hepatokines”, “FGF21”, “insulin resistance”, “inflammation”, and “lipid metabolism”. Boolean operators (AND/OR) were used to refine the search and maximize sensitivity.

The literature search primarily focused on studies published between 1998 and 2026, with particular emphasis on recent high-impact publications to ensure up-to-date evidence. However, seminal and highly cited earlier studies were also included when relevant to key mechanistic concepts. Studies were included if they addressed the epidemiological, clinical, or mechanistic relationship between MASLD and breast cancer. Studies lacking direct relevance or sufficient methodological detail were excluded.

## 3. Despite These Advances, a Critical Knowledge Gap Persists

Current evidence linking MASLD and breast cancer remains largely associative, with substantial heterogeneity in study populations, diagnostic criteria, and outcome definitions. The recent reclassification from NAFLD to MASLD—emphasizing the presence of cardiometabolic dysfunction—has further reshaped the conceptual framework, yet its implications for cancer risk stratification and mechanistic interpretation remain insufficiently defined. Moreover, the biological pathways connecting hepatic metabolic dysfunction to mammary tumorigenesis—encompassing insulin resistance, adipokine imbalance, chronic inflammation, estrogen metabolism, and hepatokine signaling—have not been comprehensively integrated into a unified pathogenic model. Importantly, it remains unclear whether MASLD represents an independent oncogenic driver or primarily reflects a surrogate marker of systemic metabolic derangement closely linked to obesity, insulin resistance, visceral adiposity, and menopausal hormonal changes. Given the substantial overlap between these metabolic abnormalities, disentangling the specific contribution of MASLD from the broader cardiometabolic environment remains challenging. This distinction is clinically relevant, as it directly impacts screening strategies, preventive interventions, and therapeutic targeting.

In this context, the aim of the present review is to examine the relationship between hepatic metabolic dysfunction and breast carcinogenesis by integrating epidemiological, clinical, and experimental evidence. Specifically, this review seeks to elucidate shared pathophysiological mechanisms and to identify potential opportunities for prevention, risk stratification, and early detection.

### 3.1. Epidemiological Evidence Linking MASLD and Breast Cancer

#### 3.1.1. Incidence Risk

Over the past decade, a growing body of epidemiological evidence has suggested that MASLD is associated with an increased incidence of breast cancer. However, because MASLD frequently coexists with obesity, insulin resistance, type 2 diabetes, and menopausal metabolic changes, it remains difficult to determine whether hepatic steatosis independently contributes to carcinogenesis or primarily reflects the broader metabolic environment underlying cancer susceptibility. Population-based and cohort studies have consistently demonstrated an association between hepatic steatosis—diagnosed through imaging modalities, serum-based indices, or clinical records—and an increased incidence of breast cancer, even after adjustment for obesity, diabetes, and other traditional metabolic risk factors.

In a large population-based cohort study from South Korea including more than seven million women, Park et al. reported that individuals with elevated fatty liver index (FLI ≥ 60) had a significantly higher risk of developing breast cancer compared with those without hepatic steatosis (HR = 1.11, 95% CI 1.05–1.17), independent of body mass index and other metabolic confounders [11]. Similarly, Kwak et al. demonstrated a higher prevalence of MASLD among patients with breast cancer compared to controls (30% vs. 20%, *p* = 0.008), with an adjusted odds ratio of up to 3.04 in non-obese women [12]. Consistent findings were reported by Nseir et al., who observed a markedly higher prevalence of hepatic steatosis in breast cancer patients compared to healthy controls (45.2% vs. 16.4%) [13].

Further supporting this association, Liu et al. demonstrated in a nationwide cohort study that earlier onset of MASLD correlates with an increased cumulative lifetime risk of developing cancer, including breast cancer, suggesting that prolonged exposure to hepatic metabolic dysfunction may amplify systemic pro-carcinogenic processes [14].

More recent evidence has reinforced the association between steatotic liver disease and extrahepatic malignancies. A 2024 large-scale meta-analysis concluded that MASLD is associated with an increased risk of several extrahepatic cancers, further supporting the concept that hepatic metabolic dysfunction may contribute to systemic carcinogenesis beyond the liver [15]. In parallel, a 2025 study reported that all subcategories of steatotic liver disease, particularly MASLD accompanied by multiple metabolic abnormalities, were associated with increased cancer risk, suggesting that the oncologic burden may rise according to the degree of metabolic derangement [16].

Breast cancer-specific data have also continued to expand. In a 2025 study published in Cell Metabolism, Li et al. reported that MASLD was associated with a higher risk of breast cancer in women with atypical hyperplasia and with poorer prognosis in patients with established breast cancer. The same study also described a historical cohort of 25,947 individuals in which breast cancer was identified as the only extrahepatic cancer significantly associated with MASLD in females and further validated this link through a meta-analysis including 11 studies with 141,273 women and 5532 breast cancer cases [17].

Interpretation of the available epidemiological evidence should be approached with caution due to substantial heterogeneity across studies. Included cohorts differ considerably in patient populations, ethnic composition, menopausal status, metabolic profiles, and breast cancer subtypes. In addition, MASLD definitions and diagnostic methods vary widely, ranging from imaging modalities and serum-based indices to administrative coding systems, potentially introducing misclassification bias. Differences in adjustment for confounding variables—including obesity, insulin resistance, diabetes, lifestyle factors, and hormonal exposure—further complicate direct comparisons across studies. Consequently, although the association between MASLD and breast cancer appears consistent, heterogeneity may influence the magnitude and interpretation of the reported risk estimates.

#### 3.1.2. Prognosis

Beyond its association with cancer incidence, MASLD has also been linked to adverse oncological outcomes. Emerging evidence suggests that hepatic metabolic dysfunction may contribute to more aggressive tumor phenotypes, increased systemic inflammation, and reduced response to therapy. Observational studies have shown that MASLD-related biomarkers—including elevated transaminases, C-reactive protein levels, and indices of insulin resistance—are associated with more advanced tumor stages and decreased disease-free survival in patients with breast cancer [18,19].

More recent data further support the prognostic relevance of steatotic liver disease in breast cancer. A 2025 study in patients with metastatic HR-positive/HER2-negative breast cancer treated with endocrine therapy and CDK4/6 inhibitors reported a high prevalence of steatotic liver disease, highlighting its potential clinical relevance in advanced disease settings and suggesting a role as a modifier of treatment response [20]. In addition, mechanistic-clinical evidence has demonstrated that MASLD is associated not only with increased breast cancer risk but also with poorer prognosis in patients with established disease, reinforcing the impact of hepatic metabolic dysfunction on tumor progression [17]. Complementing these findings, preclinical studies have shown that MASLD can directly accelerate breast tumor growth through upregulation of hepatic fibroblast growth factor 21 (FGF21), providing biological plausibility for its association with disease progression and therapeutic resistance [21].

Collectively, these findings support the hypothesis that MASLD may be associated with a systemic pro-inflammatory, endocrine, and metabolically dysregulated environment linked to tumor progression and adverse clinical outcomes. Nevertheless, most available evidence remains observational, and the independent contribution of MASLD relative to obesity, insulin resistance, and related metabolic factors remains incompletely defined.

#### 3.1.3. Special Populations

The impact of MASLD on breast cancer risk appears to vary across specific patient subgroups. Notably, the association between hepatic steatosis and breast cancer incidence is more pronounced in postmenopausal women, as initially observed by Park et al., suggesting a modulatory role of hormonal changes in the context of metabolic dysfunction [11]. More recent nationwide cohort data further support this concept, showing that although MASLD was not independently associated with overall breast cancer risk in all women, a significant association persisted in postmenopausal women with moderate obesity, reinforcing the importance of considering both menopausal status and metabolic phenotype in risk assessment [22]. The stronger associations observed in postmenopausal women may also reflect the profound metabolic and hormonal changes that accompany menopause, including increased visceral adiposity, reduced insulin sensitivity, and altered estrogen metabolism. Consequently, menopausal status may act both as a biological modifier and a confounding factor in the relationship between MASLD and breast cancer.

Additionally, the increased risk observed in non-obese women reported by Kwak et al. highlights the relevance of MASLD beyond traditional obesity-related paradigms, supporting the concept of metabolically unhealthy phenotypes independent of body mass index [12]. This observation is consistent with recent broader analyses of women’s health showing that the apparent female protection against MASLD diminishes after menopause, when visceral adiposity, insulin resistance, and extrahepatic complications become more prominent [23].

Cross-sectional clinical studies further reinforce the coexistence of both conditions. Ortiz-Pérez et al. reported that more than half of women with breast cancer evaluated by ultrasound exhibited hepatic steatosis, a prevalence higher than expected in the general population [24]. In line with this, a more recent elastography-based study found that steatotic liver disease remained highly prevalent among women with breast cancer, and that steatosis and fibrosis were more strongly associated with metabolic risk factors than with endocrine therapy exposure, emphasizing the central role of the underlying metabolic milieu in this population [25].

Ethnic and treatment-related differences may also define vulnerable subgroups. Recent data in women with early-stage hormone receptor-positive breast cancer receiving endocrine therapy showed a higher incidence and earlier onset of MASLD among Hispanic women, suggesting that ethnicity may further modify liver-related metabolic risk during breast cancer management [26].

Despite consistent epidemiological associations, several limitations temper the interpretation of these findings. Residual confounding remains a key concern, given the close overlap between MASLD and metabolic syndrome components such as obesity and insulin resistance. Heterogeneity in diagnostic criteria introduces potential misclassification bias, while the predominance of observational designs limits causal inference and raises the possibility of reverse causality. Additionally, selection bias and inconsistent reporting of key modifiers—such as menopausal status, ethnicity, and hormonal exposure—contribute to variability across studies. Therefore, although the association is robust, it remains unclear whether MASLD acts as an independent causal factor or as a surrogate marker of systemic metabolic dysfunction [22].

#### 3.1.4. MASLD Severity and Stage-Specific Oncologic Implications

MASLD should not be interpreted as a single homogeneous condition, as different disease stages may carry distinct oncologic implications. Simple steatosis is primarily characterized by hepatic lipid accumulation and metabolic dysfunction, whereas metabolic dysfunction-associated steatohepatitis (MASH) involves hepatocellular injury, oxidative stress, immune activation, and inflammatory cytokine release. Advanced fibrosis and cirrhosis represent later stages of sustained tissue remodeling, extracellular matrix deposition, vascular dysfunction, and chronic immune activation, which may reflect a more severe systemic pro-inflammatory and pro-tumorigenic phenotype. In large biopsy-confirmed cohorts, cancer risk appears to vary according to histologic severity, with fibrosis stage being one of the strongest predictors of adverse liver-related and extrahepatic outcomes [27].

Recent population-based data further support the relevance of disease severity. Peng et al. reported that cancer risk increased across subcategories of steatotic liver disease, particularly when MASLD was accompanied by multiple metabolic abnormalities, suggesting that oncologic risk may rise with the cumulative burden of metabolic dysfunction [16]. Similarly, studies evaluating steatotic liver disease phenotypes indicate that more severe phenotypes, including those with fibrosis or overlapping alcohol-metabolic injury, may be associated with higher risks of hepatic and extrahepatic outcomes than uncomplicated MASLD [28].

From a mechanistic perspective, this stage-specific distinction is clinically relevant for breast cancer. Simple steatosis may mainly reflect insulin resistance, dyslipidemia, and altered hepatokine secretion, whereas MASH and fibrosis are more strongly associated with systemic inflammation, oxidative stress, immune remodeling, and extracellular matrix-related signaling. These processes may differentially influence breast tumor biology, treatment response, and prognosis. However, most available breast cancer studies classify MASLD using imaging, serum indices, or administrative codes and do not stratify patients by steatosis severity, steatohepatitis, or fibrosis stage. Future studies should therefore incorporate fibrosis scores, elastography, histologic data when available, and standardized MASLD staging to clarify whether advanced MASLD phenotypes have stronger or distinct associations with breast cancer incidence and outcomes.

### 3.2. Mechanistic Framework Between MASLD and Breast Cancer

**Figure 1:**
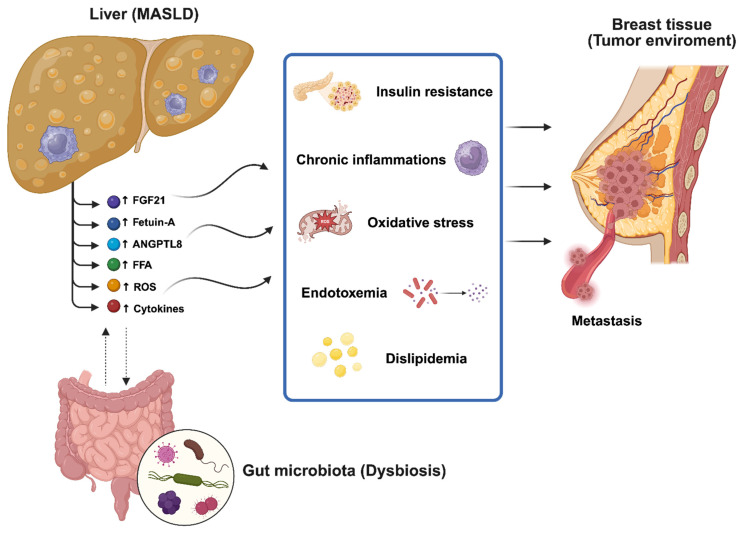
The MASLD–Breast Cancer Axis: Integrated Mechanistic Overview. FGF21, fibroblast growth factor 21; ANGPTL8, angiopoietin-like protein 8; FFA, free fatty acid; ROS, reactive oxygen species. Created in BioRender. Duart, G. (2026). https://BioRender.com/d4un2ib (accessed on 3 May 2026).

The observed association between MASLD and breast cancer is biologically plausible and supported by multiple interconnected metabolic and inflammatory pathways. Rather than acting as an isolated hepatic condition, MASLD reflects a state of global metabolic dysregulation that extends beyond the liver and influences distant organs through endocrine and paracrine signaling pathways. Central features of MASLD—including insulin resistance, chronic low-grade inflammation, dysregulated lipid metabolism, and altered hepatokine secretion—create a systemic environment that is permissive to tumor initiation, progression, and therapeutic resistance (Table 1 and Figure 1).

### 3.3. Insulin Axis

Insulin resistance, a central hallmark of MASLD, leads to compensatory hyperinsulinemia. Elevated insulin levels exert mitogenic and anti-apoptotic effects through activation of insulin receptors and insulin-like growth factor-1 receptors (IGF-1R), promoting key oncogenic signaling pathways such as PI3K/AKT/mTOR and MAPK/ERK, which drive cellular proliferation and survival [11,29]. More recent evidence has refined this concept by showing that insulin can directly stimulate breast cancer growth through coordinated activation of the insulin receptor, IGF-1R, and hybrid IR/IGF-1R receptors, supporting the notion that hyperinsulinemia amplifies signaling redundancy within the IGF axis [30]. In addition, recent data indicate that IGF-1R signaling contributes not only to tumor proliferation and survival, but also to adhesion dynamics and treatment sensitivity, further expanding its role in breast cancer progression [31]. These findings strengthen the biological plausibility of the insulin axis as a mechanistic bridge between MASLD-related metabolic dysfunction and breast carcinogenesis and suggest that dual targeting of insulin/IGF signaling may have therapeutic relevance in selected tumors [30].

Interestingly, these mechanistic pathways are not unique to breast cancer. Similar oncogenic mechanisms have also been proposed in other obesity- and metabolism-associated malignancies, including bladder cancer. Recent evidence suggests that insulin resistance in MASLD may fuel carcinogenesis through at least three overlapping mechanisms: hyperinsulinemia-driven activation of the IGF-1 signaling axis, reduction in sex hormone–binding globulin with subsequent sex steroid excess, and the establishment of a chronic pro-inflammatory and oxidative microenvironment. These mechanistic pillars have likewise been implicated in bladder cancer development and progression, reinforcing the concept that MASLD may act as part of a broader systemic oncogenic-metabolic network rather than an organ-specific condition alone [32].

Beyond its direct mitogenic effects, hyperinsulinemia may also influence the tumor microenvironment by promoting anabolic metabolism, adipocyte–tumor crosstalk, and immune suppression. Insulin/IGF signaling can enhance tumor cell nutrient uptake and survival while indirectly favoring a microenvironment enriched in inflammatory adipokines, tumor-associated macrophages, and reduced antitumor immune surveillance. These effects may be particularly relevant in metabolically unhealthy patients, in whom insulin resistance coexists with systemic inflammation and altered immune cell metabolism [33,34].

### 3.4. Hormonal Axis

Hyperinsulinemia also reduces circulating levels of sex hormone-binding globulin (SHBG), thereby increasing the bioavailability of free estrogens and androgens. This hormonal imbalance enhances proliferative signaling in breast epithelial tissue, particularly in luminal subtypes, and is especially relevant in postmenopausal women [35,36]. In parallel, adipose tissue—particularly in the context of obesity and MASLD—acts as a major extragonadal source of estrogen through aromatization of androgens. This contributes to a chronic hyperestrogenic state that promotes cell proliferation, oxidative stress, and activation of oncogenes such as c-Myc and Cyclin D1 [37,38]. Moreover, impaired hepatic function in MASLD alters estrogen metabolism and clearance, prolonging estrogen half-life and amplifying its carcinogenic effects [39].

Recent evidence further supports this axis by showing that obesity-associated insulin resistance is accompanied by reduced hepatic SHBG production and increased aromatase expression in breast adipose stromal cells, thereby reinforcing estrogen-dependent signaling in hormone receptor-positive tumors [40]. In addition, a 2024 presurgical trial in postmenopausal women with estrogen receptor-positive breast cancer reported that obesity may influence endocrine biomarker modulation during exemestane treatment, supporting the concept that excess adiposity can alter systemic sex-steroid dynamics even during aromatase inhibition [41]. Consistent with this, recent clinical data indicate that obesity is associated with a higher risk of recurrence among postmenopausal patients with hormone receptor-positive breast cancer treated with aromatase inhibitors, suggesting that the hormonal consequences of metabolic dysfunction may also have therapeutic implications [42].

Hormonal dysregulation may also interact with immune signaling within breast tissue. Estrogen receptor activation can modulate cytokine production, macrophage recruitment, and immune checkpoint-related pathways, potentially contributing to an immune-permissive microenvironment in hormone receptor-positive tumors. In postmenopausal women with MASLD, increased visceral adiposity, local aromatase activity, and altered estrogen clearance may therefore converge with inflammatory and immune pathways to influence tumor progression and endocrine therapy response [43,44].

Beyond estrogenic signaling, steroid hormones such as glucocorticoids and aldosterone may also contribute to MASLD progression and its potential oncometabolic consequences. Glucocorticoids can promote hyperglycemia, hyperinsulinemia, hepatic insulin resistance, adipose tissue lipolysis, increased hepatic de novo lipogenesis, and reduced fatty acid β-oxidation, thereby favoring hepatic steatosis and MASLD progression [45]. Aldosterone, through mineralocorticoid receptor activation, has also been implicated in hepatic inflammation, fibrosis, oxidative stress, and metabolic dysfunction, with SGK1 emerging as a key downstream effector in steroid-dependent MASLD [46].

Serum- and glucocorticoid-regulated kinase 1 (SGK1) is transcriptionally induced by glucocorticoids and mineralocorticoids and is also functionally connected to insulin/PI3K signaling, positioning it at the intersection of steroid signaling and metabolic regulation [47]. Experimental evidence suggests that increased SGK1 activity may exacerbate diet-induced obesity, insulin resistance, hepatic steatosis, and fibrotic responses, supporting its potential role as a mediator of metabolic liver injury [48].

Importantly, SGK1 is also relevant to breast cancer biology. SGK1 has been described as a tumor-promoting kinase involved in cell survival, proliferation, apoptosis resistance, and therapy resistance, including resistance to AKT inhibitors and chemotherapy in breast cancer models [49]. Therefore, SGK1 may represent a biologically plausible molecular bridge linking steroid-dependent metabolic liver dysfunction, insulin resistance, inflammation, and breast cancer progression. However, direct evidence connecting SGK1-mediated MASLD with breast carcinogenesis remains limited, and future studies should evaluate whether SGK1 expression or activity identifies metabolically vulnerable breast cancer subgroups with coexisting MASLD.

### 3.5. Inflammation

MASLD is also characterized by a state of chronic low-grade inflammation driven by activation of hepatic Kupffer cells and sustained release of pro-inflammatory cytokines, including interleukin-6 (IL-6), interleukin-1β (IL-1β), tumor necrosis factor-alpha (TNF-α), and C-reactive protein. These mediators exert systemic effects by activating pro-inflammatory signaling pathways such as NF-κB and JAK/STAT3 in distant tissues, including the breast, thereby promoting angiogenesis, tumor invasion, immune evasion, and therapeutic resistance [19,50,51,52]. Recent evidence further strengthens this inflammatory axis. In MASLD, persistent hepatic inflammation is increasingly recognized as a driver of extrahepatic carcinogenic signaling, with IL-6 and TNF-α emerging as key systemic mediators linking liver immune activation to tumor-promoting pathways [53]. In breast cancer, newer data show that IL-6/STAT3 signaling is not only associated with metastasis but also with endocrine and CDK4/6 inhibitor resistance, supporting its role in disease progression and treatment failure [54]. In addition, recent experimental work in triple-negative breast cancer has shown that an IL-6/NF-κB/STAT3 signaling loop promotes cancer stemness and chemoresistance, further supporting the relevance of systemic inflammatory activation in aggressive breast tumor phenotypes [55].

MASLD-related inflammation may further modulate the breast tumor microenvironment by favoring recruitment and activation of tumor-associated macrophages, myeloid-derived suppressor cells, and other immunosuppressive populations. These cells can promote angiogenesis, extracellular matrix remodeling, epithelial–mesenchymal transition, and metastatic dissemination. At the same time, persistent activation of IL-6/JAK/STAT3, NF-κB, and TNF-α signaling may impair cytotoxic T-cell and natural killer cell activity, facilitating immune escape and therapeutic resistance. Therefore, chronic hepatic inflammation may contribute to breast cancer progression through both systemic cytokine signaling and immune remodeling of the tumor niche [56,57].

### 3.6. Oxidative Stress

In addition, hepatic lipid accumulation in MASLD induces mitochondrial dysfunction and excessive production of reactive oxygen species, which may propagate systemically and cause oxidative DNA damage in extrahepatic tissues [58,59]. In the breast microenvironment, reactive oxygen species contribute to epithelial–mesenchymal transition, thereby enhancing the invasive and metastatic potential of tumor cells [60]. Furthermore, elevated circulating free fatty acids and oxidized lipids characteristic of MASLD can activate pattern recognition receptors such as toll-like receptor 4 (TLR4), triggering pro-inflammatory cascades that further promote tumor proliferation and resistance to apoptosis [61]. Recent indexed evidence further strengthens this link. A 2025 review highlighted that oxidative stress in breast cancer contributes not only to DNA damage and genomic instability, but also to metastatic progression through redox-sensitive signaling pathways [62]. In parallel, recent studies on tumor lipid reprogramming indicate that excess fatty acids promote invasion, metastasis, and therapeutic resistance, consistent with the dyslipidemic environment associated with MASLD [63]. Moreover, a 2025 experimental study demonstrated that linoleic acid enhances breast cancer cell migration and invasion through TLR4-dependent signaling, accompanied by focal adhesion kinase activation and increased metalloproteinase 9 (MMP-9) secretion, thereby providing direct biological support for the pro-invasive effects of circulating lipids on breast cancer cells [64].

Oxidative stress may also affect immune–tumor interactions. Excess reactive oxygen species can promote immunogenic stress signals, but sustained oxidative injury may impair cytotoxic immune cell function, enhance regulatory immune phenotypes, and support stromal remodeling. In the breast tumor microenvironment, ROS-driven lipid peroxidation products may activate macrophages, fibroblasts, and endothelial cells, thereby reinforcing angiogenesis, extracellular matrix remodeling, and invasive behavior [65]. This suggests that MASLD-associated oxidative stress may contribute not only to DNA damage and EMT, but also to immune dysregulation within the tumor niche.

### 3.7. Gut–Liver Axis

Gut microbiota dysbiosis, frequently associated with MASLD, increases intestinal permeability and circulating levels of bacterial lipopolysaccharide (LPS), contributing to metabolic endotoxemia and persistent hepatic inflammation [66,67]. Experimental evidence suggests that LPS activates TLR4 signaling in breast cancer cells, enhancing migration, invasion, and matrix MMP expression via pathways such as PI3K/AKT/GSK3β/β-catenin [68,69]. Additionally, specific gut bacteria with β-glucuronidase activity can deconjugate estrogens, increasing their systemic availability and reinforcing hormonal stimulation of breast tissue [70]. Recent reviews further support that gut microbial dysbiosis may influence breast cancer progression not only through estrogen metabolism, but also by modulating host immunity, inflammatory tone, and therapeutic response [71]. Prospective and longitudinal studies have also suggested that endocrine therapy is associated with measurable shifts in gut microbiota composition and metabolomic profiles in women with breast cancer, reinforcing the dynamic interaction between systemic metabolism, treatment exposure, and the intestinal ecosystem. Moreover, recent work on the estrobolome has highlighted microbial estrogen-processing pathways as plausible mechanistic nodes linking dysbiosis, altered estrogen recirculation, and hormone-dependent breast carcinogenesis [72]. Collectively, these mechanisms support the existence of a functional “gut–liver–breast axis,” in which hepatic metabolic dysfunction acts as a central regulator of systemic processes that facilitate breast carcinogenesis [71].

Gut dysbiosis may additionally influence antitumor immunity by altering microbial metabolites, systemic endotoxin exposure, and immune cell priming. Increased LPS translocation can activate TLR4-dependent signaling in both tumor and immune cells, promoting inflammatory macrophage activation, immunosuppressive myeloid expansion, and impaired adaptive immune responses. Microbiota-derived metabolites may also affect T-cell differentiation, natural killer cell activity, and response to systemic cancer therapies [73,74,75]. Therefore, the gut–liver axis may act as an upstream regulator of the breast tumor immune microenvironment.

### 3.8. Hepatokines and Endocrine Crosstalk

#### FGF21: A Potential Hepatic–Breast Axis

FGF21, a key hepatokine involved in energy homeostasis, has emerged as a potential molecular mediator linking hepatic dysfunction to breast cancer progression. Circulating levels of FGF21 are significantly elevated in MASLD, reflecting underlying metabolic stress and a state of functional resistance to its action [76,77]. Although initially considered a compensatory and protective response, chronic overexpression of FGF21 appears to indicate a state of hormonal resistance analogous to insulin resistance [77,78] (Figure 1).

Preclinical evidence suggests a possible role for FGF21 in tumor progression, although current data remain largely experimental. Sui et al. demonstrated in murine models that MASLD induces hepatic overexpression of FGF21, leading to elevated circulating levels that accelerate breast tumor growth. Mechanistically, exogenous FGF21 promotes breast cancer cell survival by activating anti-apoptotic signaling pathways, including STAT3 and AKT/FoxO1, and reduces chemotherapy-induced cytotoxicity, particularly in response to doxorubicin. Conversely, genetic ablation of FGF21 attenuated pro-tumorigenic effects in experimental models, supporting a potential mechanistic association between this hepatokine and breast cancer progression [21].

Preliminary clinical studies further support the potential relevance of FGF21 in this context. Patients with breast cancer exhibit significantly higher serum FGF21 levels compared to healthy controls, with a proposed diagnostic threshold of approximately 130 pg/mL identifying nearly two-thirds of patients as “FGF21-positive.” Moreover, elevated FGF21 levels at diagnosis have been associated with reduced overall survival in preliminary studies, although prospective validation remains lacking [79]. Notably, interventions that improve metabolic status, such as adjuvant endocrine therapy, have been shown to reduce circulating FGF21 levels in parallel with improvements in metabolic parameters, including glycemic control [80].

Recent indexed evidence further highlights the possible biological and translational relevance of this axis. A 2025 review emphasized that FGF21 occupies a paradoxical position in MASLD, acting as a hepatoprotective and anti-steatotic factor while, under chronic metabolic stress, also behaving as a marker of FGF21 resistance and persistent systemic dysfunction, which may help explain its association with unfavorable oncologic contexts [81]. In parallel, a 2025 review of FGF/FGFR signaling in breast cancer highlighted that fibroblast growth factor pathways are increasingly recognized as regulators of breast cancer metabolic reprogramming, tumor survival, and therapy resistance, providing broader biological context for the pro-tumorigenic effects attributed to FGF21 [82]. Additional recent clinical data have linked circulating FGF21 to adverse body composition and biomarker profiles after breast cancer diagnosis, suggesting that this hepatokine may reflect broader metabolic vulnerability in affected patients [83].

Despite these findings, the relationship between FGF21 and specific tumor characteristics remains incompletely understood. Some studies have not identified significant associations between FGF21 levels and receptor status (ER, PR, HER2), suggesting that FGF21 may primarily reflect systemic metabolic dysfunction rather than tumor-intrinsic biology [79]. Future studies incorporating stratification by tumor subtype and MASLD status are needed to determine whether FGF21 identifies metabolically vulnerable subgroups, such as postmenopausal women with luminal tumors and coexisting MASLD.

Importantly, elevated FGF21 levels may reflect systemic metabolic stress rather than a liver-specific oncogenic signal and therefore should be interpreted within the broader context of metabolic dysfunction.

### 3.9. Emerging Hepatokines: Fetuin-A and ANGPTL8 in the MASLD–Breast Cancer Axis

Beyond FGF21, additional hepatokines such as Fetuin-A and angiopoietin-like protein 8 (ANGPTL8) have emerged as potential mediators linking hepatic metabolic dysfunction with breast carcinogenesis.

Fetuin-A, a hepatocyte-derived glycoprotein elevated in MASLD and insulin resistance, has been proposed as a potential regulator of metabolic inflammation. Recent studies demonstrate that Fetuin-A acts as an endogenous ligand of TLR4, promoting macrophage activation and systemic low-grade inflammation, thereby contributing to a pro-tumorigenic microenvironment [84]. In oncologic contexts, Fetuin-A has been shown to facilitate tumor cell adhesion, migration, and extracellular matrix remodeling, processes that are critical for metastasis [85]. Moreover, recent clinical observations suggest that elevated circulating Fetuin-A levels may be associated with obesity-related cancers, including breast cancer, although its clinical utility as a biomarker remains uncertain [86].

ANGPTL8, another hepatokine involved in lipid and glucose homeostasis, has also gained attention due to its role in metabolic reprogramming. ANGPTL8 regulates triglyceride metabolism through inhibition of lipoprotein lipase and is upregulated in MASLD and related metabolic disorders [87]. Recent experimental studies suggest that ANGPTL8 and related ANGPTL family members may contribute to tumor progression by influencing lipid availability, angiogenesis, and tumor microenvironment remodeling [88,89]. In addition, emerging data suggest that ANGPTL8 may influence cancer cell metabolic flexibilityand adaptation to nutrient stress, although direct evidence in breast cancer remains limited [90].

Collectively, these hepatokines support the emerging concept that the liver may influence systemic oncogenic signaling through endocrine and metabolic pathways. Fetuin-A and ANGPTL8 may represent additional components of the gut–liver–tumor axis linking metabolic dysfunction with inflammation, lipid metabolism, and cancer progression, although their precise clinical relevance remains to be established. However, their specific roles in breast cancer remain incompletely defined, and further mechanistic and clinical studies are required to determine their utility as biomarkers or therapeutic targets. (Figure 1).

## 4. Therapeutic and Clinical Implications

Preclinical models have been instrumental in elucidating how the metabolic milieu associated with MASLD promotes breast cancer development and progression, particularly in aggressive subtypes such as triple-negative breast cancer. Triple-negative breast cancer exhibits a pronounced dependence on fatty acid oxidation (FAO) to sustain its energy demands and survival. Overexpression of key FAO-related enzymes, such as carnitine palmitoyltransferase 1A (CPT1A), has been associated with increased tumor aggressiveness and poorer clinical outcomes [91,92]. This metabolic phenotype appears to be further exacerbated by the lipotoxic environment characteristic of MASLD. Experimental models have demonstrated that high-fat diets and induced hyperlipidemia significantly enhance tumor growth and metastatic potential in breast cancer [93,94]. These findings suggest that excess circulating free fatty acids derived from hepatic steatosis may serve as a metabolic substrate that fuels tumor progression.

More recent original studies further support the therapeutic relevance of targeting FAO in breast cancer. In HER2-positive models, genetic or pharmacologic inhibition of CPT1A delayed tumor onset, reduced metastatic burden, and enhanced the efficacy of anti-HER2 therapy, partly through remodeling of the tumor immune microenvironment, supporting FAO blockade as a rational combinatorial strategy beyond triple-negative breast cancer [95]. In estrogen receptor-positive breast cancer, activation of FAO has also been implicated in tamoxifen resistance; targeting c-Jun suppressed FAO and restored endocrine sensitivity in resistant cells, suggesting that metabolic interventions may also help overcome treatment resistance in luminal disease [96].

From a clinical perspective, recent observational data indicate that steatotic liver disease is highly prevalent among patients with metastatic HR-positive/HER2-negative breast cancer receiving endocrine therapy plus CDK4/6 inhibitors, highlighting the need to better characterize how underlying liver-metabolic dysfunction may influence treatment tolerance and long-term outcomes in advanced disease [20]. In parallel, emerging clinical evidence suggests that circulating FGF21 may capture a broader state of metabolic vulnerability after breast cancer diagnosis, as its levels have been associated with adverse body composition and metabolic biomarker profiles during follow-up [83].

Collectively, these findings position lipid metabolism—and mediators such as FGF21—as promising therapeutic and clinical targets. Pharmacological inhibition of FAO, particularly in combination with systemic anticancer therapies, may represent a potential adjuvant strategy to sensitize tumors to treatment in metabolically vulnerable patients. Likewise, biomarkers linked to hepatic-metabolic dysfunction may help refine risk stratification and monitoring. However, prospective clinical studies are still required to determine whether interventions targeting MASLD-related metabolic pathways can translate into improved oncological outcomes.

In addition to tumor-directed metabolic strategies, interventions targeting MASLD and systemic metabolic health may represent an important future direction in breast cancer care. Weight loss and structured lifestyle interventions have demonstrated feasibility and favorable metabolic effects in breast cancer survivors with overweight or obesity, including improvements in weight, adipokine profiles, physical activity, and quality of life, although definitive effects on recurrence and survival remain under investigation. The Breast Cancer Weight Loss trial showed that a remotely delivered weight-loss intervention achieved clinically meaningful weight reduction at 12 months in women with breast cancer and elevated body mass index, supporting the feasibility of integrating metabolic interventions into oncology care [97]. Similarly, lifestyle trials such as WISER Survivor and LEAN Self-Guided have shown that exercise- and diet-based interventions can improve body composition and metabolic health in breast cancer survivors, providing a rationale for testing whether these changes translate into improved oncologic outcomes [98,99]. From a hepatometabolic perspective, GLP-1 receptor agonists are of particular interest because randomized trials have shown that semaglutide reduces liver steatosis in patients with NAFLD/MASLD, while emerging observational data suggest potential benefits of GLP-1-based therapies in MASLD-related outcomes [100]. However, whether GLP-1 receptor agonists or other MASLD-directed therapies can modify breast cancer recurrence, treatment tolerance, or survival remains unknown and should be evaluated in prospective studies specifically designed for patients with coexisting breast cancer and MASLD.

## 5. Future Directions

Despite the growing body of epidemiological, mechanistic, and translational evidence linking MASLD with breast cancer, several critical gaps remain that warrant further investigation. First, there is a need for well-designed prospective longitudinal studies to establish causality and to disentangle whether MASLD represents an independent oncogenic driver or a surrogate marker of systemic metabolic dysfunction. Future research should incorporate standardized definitions of MASLD, including stratification by disease severity (simple steatosis, steatohepatitis, and fibrosis), to better define its differential impact on breast cancer incidence, progression, and survival outcomes.

Second, mechanistic studies should aim to further elucidate the integrative crosstalk between metabolic, hormonal, inflammatory, and hepatokine-mediated pathways. In particular, the role of hepatokines such as FGF21, Fetuin-A, and ANGPTL8 requires deeper investigation in human cohorts, including their interaction with tumor subtype, menopausal status, and treatment exposure. Multi-omics approaches integrating metabolomics, transcriptomics, and microbiome profiling may provide a more comprehensive understanding of the proposed gut–liver–breast axis and its contribution to carcinogenesis.

Third, translational research should prioritize the identification and validation of clinically applicable biomarkers. Circulating FGF21 and other metabolic mediators hold promise for risk stratification, early detection, and monitoring of therapeutic response; however, their clinical utility remains to be confirmed in large-scale prospective studies.

Finally, interventional clinical trials are urgently needed to evaluate whether targeting metabolic dysfunction can modify breast cancer risk or improve oncological outcomes. Strategies aimed at improving insulin sensitivity, reducing hepatic steatosis, modulating lipid metabolism (e.g., FAO inhibition), or targeting inflammatory pathways may represent novel adjuvant approaches, particularly in metabolically vulnerable populations. The integration of metabolic and hepatic assessment into routine oncologic care may ultimately enable a more personalized and preventive approach to breast cancer management.

## 6. Conclusions

The growing body of epidemiological, experimental, and translational evidence reviewed in this article supports a biologically plausible association between metabolic dysfunction-associated steatotic liver disease (MASLD) and breast cancer. Although current data remain insufficient to establish a direct causal relationship, MASLD appears to coexist with a systemic metabolic environment characterized by insulin resistance, chronic inflammation, oxidative stress, dysregulated lipid metabolism, hormonal imbalance, gut microbiota alterations, and immune remodeling, all of which may contribute to tumor initiation, progression, and therapeutic resistance. Importantly, many of these pathways converge on key oncogenic signaling networks, including PI3K/AKT/mTOR, JAK/STAT3, NF-κB, and metabolic reprogramming pathways involved in fatty acid oxidation and cellular survival.

Emerging evidence also suggests that hepatokines such as FGF21, Fetuin-A, and ANGPTL8 may participate in the complex crosstalk between hepatic dysfunction and breast tumor biology. However, their precise mechanistic role and clinical utility remain largely investigational, and further validation in prospective human studies is required. Similarly, increasing recognition of immune modulation, tumor microenvironment remodeling, and gut–liver–breast axis interactions highlights the need to interpret MASLD not only as a hepatic disorder, but as part of a broader systemic immunometabolic disease spectrum with potential oncologic implications.

An important limitation of the currently available literature is the substantial heterogeneity in study design, MASLD diagnostic criteria, fibrosis assessment, patient populations, and adjustment for confounding metabolic variables such as obesity, insulin resistance, and menopausal status. In addition, most available studies evaluate MASLD as a single entity, despite the likelihood that steatosis, steatohepatitis, and advanced fibrosis represent biologically distinct phenotypes with potentially different oncologic consequences. Future studies incorporating standardized MASLD staging, fibrosis stratification, molecular profiling, and tumor subtype characterization will therefore be essential to better define these relationships.

From a translational perspective, the integration of metabolic and hepatic assessment into breast cancer risk stratification may represent an important future clinical direction. Identification of metabolically vulnerable patients through hepatometabolic biomarkers, imaging-based liver phenotyping, inflammatory profiling, and assessment of insulin resistance could eventually support more personalized preventive and therapeutic strategies. Moreover, interventions targeting systemic metabolic dysfunction—including lifestyle modification, weight reduction, exercise-based programs, insulin-sensitizing therapies, and emerging hepatometabolic agents such as GLP-1 receptor agonists—may have the potential to complement conventional oncologic treatment, particularly in patients with coexisting MASLD and breast cancer.

Ultimately, prospective longitudinal cohorts, mechanistic translational studies, and interventional clinical trials are needed to determine whether improving hepatic and metabolic health can favorably influence breast cancer incidence, treatment response, recurrence, and survival. A deeper understanding of the MASLD–breast cancer axis may help establish a more integrated metabolic-oncology framework, bridging hepatology, endocrinology, immunology, and cancer medicine toward more personalized patient care.

## Figures and Tables

**Table 1:** Shared Pathophysiological Mechanisms Linking MASLD and Breast Cancer. IGF-1, insulin-like growth factor-1; IR, insulin resistance; IGF-1R, insulin-like growth factor-1 receptors; PI3K, phosphoinositide 3-kinase; AKT, Protein Kinase B; mTOR, mechanistic target of rapamycin; MAPK, mitogen-activated protein kinase; ERK, extracellular signal-regulated kinase; SHBG, sex hormone-binding globulin; IL-6, interleukin-6; IL-1β, interleukin-1 beta; TNF-α, tumor necrosis factor-alpha; CRP, C-reactive protein; NF-κB, Nuclear factor kappa-light-chain-enhancer of activated B cells; JAK, janus kinase; STAT3, signal transducer and activator of transcription 3; ROS, reactive oxygen species; DNA, deoxyribonucleic acid; EMT, Epithelial-to-Mesenchymal Transition; LPS, lipopolysaccharide; TLR4, toll-like receptor 4; MMP, metalloproteinase; FGF21, fibroblast growth factor 21; *FoxO1*, Forkhead box O1, ANGPTL8, angiopoietin-like protein 8.

Pathway	Key Mediators	Oncogenic Effects in Breast Tissue	Level of Evidence
Insulin resistance & hyperinsulinemia	Insulin, IGF-1, IR, IGF-1R	Activation of PI3K/AKT/mTOR and MAPK/ERK pathways → increased proliferation, survival, anti-apoptosis	Strong (epidemiological + mechanistic)
Hormonal dysregulation	Insulin, SHBG, estradiol, androgens	Increased bioavailability of sex hormones → stimulation of luminal tumor growth	Strong (clinical + mechanistic)
Adipose tissue-derived estrogen production	Aromatase, estrone, estradiol	Chronic hyperestrogenic state → activation of c-Myc, Cyclin D1, oxidative stress, proliferation	Strong (mechanistic + clinical)
Impaired hepatic estrogen metabolism	Reduced hepatic clearance, altered conjugation pathways	Prolonged estrogen exposure → enhanced carcinogenic signaling	Moderate (clinical + biological plausibility)
Chronic low-grade inflammation	IL-6, IL-1β, TNF-α, CRP	Activation of NF-κB, JAK/STAT3 → angiogenesis, invasion, immune evasion, therapy resistance	Strong (translational + clinical)
Oxidative stress & mitochondrial dysfunction	ROS, lipid peroxidation products	DNA damage, genomic instability, EMT induction → increased invasiveness and metastasis	Strong (experimental + translational)
Gut dysbiosis & endotoxemia	LPS, microbiota-derived metabolites	Systemic inflammation, TLR4 activation → increased migration, invasion, MMP expression	Moderate (experimental + emerging clinical)
Estrogen reactivation by microbiota	β-glucuronidase-producing bacteria	Increased circulating estrogens → enhanced hormonal stimulation of breast tissue	Emerging (preclinical + early clinical)
Gut–liver–breast axis integration	Combined metabolic, inflammatory, hormonal mediators	Systemic pro-tumorigenic environment → initiation and progression of breast cancer	Conceptual (integrative evidence)
FGF21 axis	FGF21, STAT3, AKT/*FoxO1*	Anti-apoptotic signaling, tumor growth acceleration, chemotherapy resistance	Strong (experimental + translational)
Fetuin-A signaling	Fetuin-A, TLR4	Macrophage activation, inflammation, tumor adhesion and metastasis	Moderate (translational + experimental)
ANGPTL8 & lipid metabolism	ANGPTL8, ANGPTL family, lipoprotein lipase	Lipid availability, angiogenesis, metabolic flexibility → tumor survival and progression	Emerging (experimental + metabolic evidence)
Liver-derived exosomal signaling	Adipocyte-oriented exosomes, lipid mediators	Inter-organ communication → promotion of primary tumor growth and systemic metabolic reprogramming	Emerging (experimental + translational)

## Data Availability

No new data were created or analyzed in this study. Data sharing is not applicable to this article.
